# Maternal exposure to benzene and toluene and preterm birth. A longitudinal study

**DOI:** 10.1590/1516-3180.2019.0224170919

**Published:** 2020-03-06

**Authors:** Djalma Antonio Almeida dos Santos, Luiz Fernando Costa Nascimento

**Affiliations:** I MSc. Doctoral Student, Postgraduate Program on Mechanical Engineering, Department of Energy, Universidade Estadual de São Paulo (UNESP), Guaratinguetá, Brazil.; II MD, PhD. Researcher, Postgraduate Program on Mechanical Engineering, Department of Energy, Universidade Estadual de São Paulo (UNESP), Guaratinguetá, Brazil.

**Keywords:** Benzene, Toluene, Air pollutants, Child health, Prenatal care, Public policy, Preterm, Premature delivery, Neonatal mortality

## Abstract

**BACKGROUND:**

Exposure to air pollutants has several effects on human health, including during pregnancy.

**OBJECTIVE:**

To identify whether exposure to benzene and toluene among pregnant women contributes to preterm delivery.

**DESIGN AND SETTING:**

Longitudinal study using data on newborns from mothers living in São José dos Campos (SP) in 2016, who had been exposed to benzene and toluene.

**METHODS:**

A logistic regression model with three hierarchical levels was constructed using maternal variables relating to newborns, and using benzene and toluene concentrations in quartiles. Occurrences of cesarean births, twins or malformations were excluded. Maternal exposure windows of 5, 10, 15, 30, 60 and 90 days prior to delivery were considered.

**RESULTS:**

Out of the 9,562 live births, 3,671 newborns were included and 343 newborns were born at less than 37 weeks of gestation (9.3%). The average birth weight was 3,167.2 g. Exposure to benzene and toluene was significantly associated (P = 0.04) with preterm delivery in the five-day window. There was no association in any of the other exposure windows.

**CONCLUSIONS:**

It was possible to identify that maternal exposure to benzene and toluene has an acute effect on preterm delivery.

## INTRODUCTION

Preterm birth is defined as birth before 37 full weeks of gestation and is associated with higher neonatal morbidity and mortality,^1^ along with diseases resulting from prematurity. The effects from preterm birth may extend into adulthood.^2^

In a multicenter study conducted in Brazil, the prematurity rate was estimated to be 11.7%. However, the rate reported through the national information system for live births (SINASC) was 7.1%.^3^

Many factors have been associated with preterm delivery, including pregnancies at ages of below 19 and above 34 years, low maternal schooling level, low number of prenatal visits, twin pregnancy, shortened cervix, premature rupture of the amniotic membrane, inflammation and recent exposure to environmental pollutants.^4^^,^^5^

Carbon monoxide (CO), ozone (O_3_), nitrogen dioxide (NO_2_) and sulfur dioxide (SO_2_) are the gaseous pollutants that have been most studied.^6^ Particulate matter with an aerodynamic diameter of less than 10 µm (PM_10_) has also been investigated. This is composed of a mixture of solid and liquid particles that adsorb polycyclic aromatic hydrocarbons and ions such as sulphates, nitrates and metals.^7^

Besides these, reports have also shown that associations with benzene and toluene exist. These substances are emitted mainly by the petrochemical industry, but also come from the vehicular fleet. It has not been defined whether their effects occur in an acute or chronic manner, or what the mechanism of action for these effects might be. However, it is known that the action of air pollutants is associated with the pro-oxidant effects of lipids and proteins, along with formation of free radicals, which generate oxidative stress and inflammation.^8^^,^^9^^,^^10^^,^^11^

## OBJECTIVE

The objectives of this study were to identify the possible effects of maternal exposure to benzene and toluene at the onset of preterm delivery and to evaluate whether these are acute or chronic.

## METHODS

This was a longitudinal study that used data on live births from pregnant women living in São José dos Campos (SP), Brazil, in 2016. This municipality has a population of approximately 700,000 inhabitants. It is a regional center with several industries, including automobile, aerospace and petrochemical industries, among which the Petrobras oil refinery stands out. Via Dutra, the busiest highway in Brazil, connecting the metropolitan areas of São Paulo and Rio de Janeiro, crosses this municipality.

Data were obtained from birth certificates, i.e. declarations of live birth (Declaração de Nascido Vivo, DNV) that are recorded in the live birth information system (Sistema de Informação sobre Nascidos Vivos, SINASC).^12^ Newborns weighing less than 500 grams, twin and trigeminal births and fetuses with congenital malformations were all excluded from this study because these occurrences are possibly associated with preterm birth. Cesarean deliveries were also excluded because of the possibility of iatrogenic prematurity.

Data on the pollutants benzene and toluene were obtained from the database of the Environmental Company of the State of São Paulo (Companhia Ambiental do Estado de São Paulo, CETESB), which is in the public domain.^13^

Statistical analysis was performed using a hierarchical logistic regression model. The pollutants were divided into quartiles and considered in relation to the first quartile (reference category).

The dependent variable was the presence of preterm birth. The independent variables were classified into three levels: distal, intermediate and proximal. The classification of the variables as such is described below.

The distal level included maternal age, marital status and schooling level. Maternal age was categorized as favorable when it was within the range from 20 to 34 years; or as unfavorable when it was 19 years or younger or 35 years or older, i.e. with greater risk of preterm birth. Marital status was categorized as single, separated or widowed (unfavorable); or as married or in a stable union (favorable). Schooling level was classified as elementary and high school only (unfavorable); or as tertiary-level, i.e. university or technical college (favorable).

The intermediate level included the number of prenatal visits, which was categorized as unfavorable when this number was between zero and six or as favorable when it was seven or more; and the time at which prenatal care began, which was categorized as favorable when this was no later than the third month or as unfavorable when this was from the fourth month onwards.

The proximal level included the sum of concentrations of the pollutants recorded in the 5, 10, 15, 30, 60 and 90-day windows prior to childbirth.

The pollutants benzene and toluene were analyzed in terms of daily averages, in µg/m^3^. For analysis purposes, the sums of the concentrations in the 5, 10, 15, 30, 60 and 90-day windows before childbirth were considered. The aim of this procedure was to assess whether the cumulative effects of these pollutants on the onset of preterm delivery occurred in an acute or a chronic manner. Bivariate analysis was performed on the variables within the three levels, separately. At the distal level, variables with a P-value ≤ 0.20 were included in the multivariate analysis and variables that presented P-value < 0.05 at this level were maintained at the next phase of the analysis.

The same procedure was performed for intermediate-level variables, and then those with P-value < 0.05 were adjusted according to the variables of the previous level. At that moment, the hierarchical model contained two levels of variables: distal and intermediate. Lastly, for each of the pollutant exposure windows with P-value ≤ 0.20, the variables at this level were maintained for each window with P-value < 0.05, thus completing the three levels of this analysis.

For the exposure windows that continued to present P-values < 0.05 in the final model and thus were retained in the model, we also performed a χ^2^ trend test to identify any possible dose-response effect.

For the statistical analysis, we used the Epi Info software, version 7.2. The significance level used in the analyses was alpha = 5%.

Because this study used data that are available online with public access, and it was impossible to identify the subjects analyzed, the study was not submitted to a research ethics committee.

## RESULTS

Out of the total number of 9,562 live births in 2016 in São José dos Campos, Brazil, 3,671 (38.0%) that met the inclusion criteria were analyzed. There were 1,826 male newborns (50.9%) and 1,845 female newborns (49.1%). Their mean birth weight was 3,167.2 g (standard deviation, SD = 495.0 g). Among the live births that met the inclusion criteria, 343 (9.3%) were found to have taken place at a gestational age of less than 37 weeks.

The mean daily levels and respective standard deviations (SD) for the pollutants were as follows: benzene 6.56 µg/m^3^ (SD = 4.83); and toluene 21.38 µg/m^3^ (SD = 16.66).

The results from the bivariate analysis on the distal and intermediate levels showed that only the number of prenatal care consultations was significant (P-value < 0.01). These results are shown in Table 1.

**Table 1.** Descriptive analysis on distal and intermediate variables^#^. São José dos Campos (SP), 2016

Variable	Categories	Delivery at < 37 weeks (n)	Delivery at ≥ 37 weeks (n)	P-value
Age	20 to 34	2,128	6,371	0.61
	< 20 and > 34	265	763	
Marital status	Married	2651	5,799	0.54
	Unmarried	331	693	
Schooling	Tertiary-level	321	706	0.85
	High school	2,675	5,806	
Prenatal consultations	7 or more	1,357	7,136	< 0.01
	< 7	430	596	
Prenatal start	Not later than 3^rd^ month	872	7,266	0.90
	4^th^ month onwards	102	862	

^#^The differences between the total numbers of cases included in Table 1 and the total numbers of births result from lack of information about some variables.

In the final analysis, both of the pollutants were considered at the proximal level, adjusted for the number of prenatal care consultations. The odds ratios (ORs) and their respective 95% ­confidence interval (95% CI) for the accumulated 5, 10 and 15 days relating to benzene and toluene exposure, with emphasis on the accumulated five-day concentration regarding benzene (OR = 1.12; 95% CI: 1.01-1.23) and toluene (OR = 1.12; 95% CI: 1.01-1.23) are shown in Table 2.

**Table 2.** Odds ratio (OR) with 95% confidence interval (95% CI) and significance level (P-value) for maternal exposure to benzene and toluene in relation to preterm delivery, for benzene and toluene exposure windows of 5, 10 and 15 days prior to delivery. São José dos Campos (SP), 2016*

	OR	95% CI	P-value
Benzene 5 days (µg/m^3^)	1.12	1.01-1.23	0.04
Benzene 10 days (µg/m^3^)	1.07	0.97-1.19	0.16
Benzene 15 days (µg/m^3^)	1.04	0.94-1.15	0.46
Toluene 5 days (µg/m^3^)	1.12	1.01-1.23	0.04
Toluene 10 days (µg/m^3^)	1.10	0.99-1.22	0.06
Toluene 15 days (µg/m^3^)	1.07	0.97-1.19	1.18

*Adjusted for the number of prenatal consultations.

Table 3 presents accumulation data for 30, 60 and 90 days, for benzene and toluene. No significant values were found for this range of exposure.

The χ^2^ trend for the accumulated five-day periods is shown in Table 4. It shows that both benzene and toluene had significant cumulative effects.

**Table 3.** Odds ratio (OR) with 95% confidence interval (95% CI) and significance level (P-value) for maternal exposure to benzene and toluene in relation to preterm labor and delivery, for benzene and toluene exposure windows of 30, 60 and 90 days prior to delivery. São José dos Campos (SP), 2016*

Pollutant (µg/m^3^)	Window of exposure	OR	95% CI	P-value
Benzene	30 days	1.06	0.95-1.17	0.30
	60 days	1.03	0.92-1.14	0.61
	90 days	1.02	0.92-1.13	0.93
Toluene	30 days	1.09	0.99-1.21	0.07
	60 days	1.01	0.91-1.21	0.81
	90 days	0.99	0.89-1.10	0.93

*Adjusted for the number of prenatal consultations.

**Table 4.** χ² trend for accumulation of 5 days of benzene and toluene exposure, in quartiles of level of concentration (Q1, Q2, Q3 and Q4). São José dos Campos (SP), 2016*

Pollutant and time of exposure	Quartile	Number of cases	Number of controls	OR	P-value
Benzene 5 days (µg/m^3^)	Q1^#^	78	883	1.00	0.04
	Q2	84	856	1.11	
	Q3	80	753	1.20	
	Q4	101	836	1.37	
Toluene 5 days (µg/m^3^)	Q1^#^	70	801	1.00	0.05
	Q2	83	849	1.12	
	Q3	92	843	1.25	
	Q4	98	835	1.34	

*Adjusted for the number of prenatal consultations; ^#^Reference category.

## DISCUSSION

This study identified an association between preterm birth and maternal exposure to benzene and toluene five days before delivery, after adjusting for the number of consultations attended by the pregnant woman during prenatal care. Thus, a possible acute effect was found.

It is known that exposure to air pollutants is associated with several respiratory and cardiovascular diseases, as well as with preterm birth.^8^ Few studies in the literature have correlated exposure to benzene and toluene with premature delivery. In Brazil, to the best of our knowledge, this is the first study on this association.

The analysis on distal and intermediate factors (Table 1) showed that there was only an association with the number of visits made during prenatal care (P < 0.01). Pregnant women who made not more than six visits were more likely to have a premature delivery. This confirms the observations from a Brazilian multicenter study, which found similar results (OR = 2.13; 95% CI: 1.57-2.88).^5^

However, the factor of maternal age was not found to be associated with preterm birth, contrary to the findings from a Chinese study. In that study, two age groups were formed and the group older than 30 years presented higher risk of prematurity.^14^

There was also no association with maternal marital status, since women living without partners were not found to be at higher risk of preterm birth. This was discordant with the findings from an American study, which showed that those living without a partner presented significantly higher risk (relative risk, RR 10.67%; 95% CI: 10.23-11.12), in relation to those with a partner (RR = 7.21%; 95% CI: 6.92-7.50).^15^

Regarding schooling level as a risk factor for low birth weight, our findings were concordant with those from a Brazilian study carried out in the city of Rio de Janeiro, where no association between low schooling level and low birth weight (OR = 0.93) was found.^16^ However, the results from the present study were contrary to the findings from a study conducted in Spain (OR = 2.05)^17^ and to those from a Brazilian study conducted in the city of São José do Rio Preto (P < 0.01), which found strong associations between maternal schooling level and low birth weight.^18^

Other comparisons with the Brazilian literature are very difficult, given the scarcity of studies on the effect of maternal exposure to air pollutants and occurrence of premature delivery. In a single study conducted by Lima et al.,^19^ a significant association between premature delivery and exposure to PM_10_ was found on the day of delivery (lag 0), the day before delivery (lag 1) and three days prior to delivery. However, a different approach was used in their study.

Analysis on acute exposure (Table 2) showed that there were associations with premature delivery for the sum of five days prior to delivery, regarding maternal exposure to benzene (OR = 1.12; 95% CI: 1.01-1.23) and to toluene (OR = 1.12; 95% CI: 1.01-1.23). The results relating to benzene were partly concordant with findings from another study conducted in Spain, which found associations between maternal exposure to benzene and occurrence of premature delivery, from analysis on the whole gestational period, when the levels exceeded 2.7 µg/m^3^ (OR = 1.38; 95% CI: 1.03-1.84).^8^ In the present study, the acute effect of exposure was identified, but no data on the acute effect of these pollutants are available in the literature.

In a study conducted in Canada, Poirier et al. showed that there was an association between exposure of pregnant women to toluene in the second quartile of toluene concentrations (OR = 1.35; 95% CI: 1.12-1.63) and preterm delivery. In this Canadian study, exposure was considered throughout the gestational period and the findings are partially concordant with our results, in which we found an association with maternal exposure for five days.^10^

On the other hand, there was no significant association between maternal exposure to benzene and toluene over other exposure windows such as 30, 60 and 90 days.

The χ^2^ trend analysis on the five-day windows, with adjustment for the number of consultations, showed that the higher the concentrations of benzene and toluene were, the greater the chance of birth of a preterm fetus was. This highlights the possible cumulative effect of exposure.

One possible mechanism for the onset of preterm birth caused by exposure to air pollutants is thought to relate to release of cytokines and reactive oxygen species, thereby causing oxidative stress.^20^ In the case of benzene and toluene, there are no studies that explain the mechanism of action of these two pollutants in triggering preterm birth. Moreover, there are no World Health Organization (WHO) guidelines for acceptable values for benzene or toluene, unlike in relation to other air pollutants.^6^

This study had certain limitations. The mothers’ socioeconomic conditions, home address, living conditions, preexisting diseases and smoking status were not available through SINASC. The concentrations of the air pollutants were considered homogeneous throughout the city, which might not reflect the reality. Moreover, the pregnant women had been free to circulate during their pregnancies. Another possible limitation of the present study may have been its non-inclusion of other air pollutants. However, a previous analysis had not shown any correlation between these air pollutants and the outcome.

## CONCLUSIONS

Despite the limitations of the present study, it was possible to identify the deleterious effect of maternal exposure to benzene and toluene on the preterm delivery in a medium-sized city in Brazil, and to ascertain that this was an acute effect. The results presented here may be useful for public policy implementation, with the aim of reducing these concentrations.
